# A comparison of the Food and Drug Administration’s and Health Canada’s regulatory decisions about failed confirmatory trials for oncology drugs: an observational study

**DOI:** 10.1186/s40545-021-00375-y

**Published:** 2021-10-28

**Authors:** Joel Lexchin

**Affiliations:** 1 Professor Emeritus, School of Health Policy and Management, York University, 4700 Keele St., ON M3J 1P3 Toronto, Canada; 2 Department of Family and Community Medicine, University of Toronto, Toronto, Canada; 3 University Health Network, Toronto, ON Canada

**Keywords:** Accelerated approval, Confirmatory trials, Food and Drug Administration, Health Canada, Notice of Compliance with conditions, Oncology drugs

## Abstract

**Background:**

Oncology drugs are frequently approved on the basis of surrogate outcomes that require further trials to confirm the benefits, but at times these trials fail and regulators need to decide whether to withdraw approval for the indication and/or to remove the drug from the market. This study compares decisions by the Food and Drug Administration and Health Canada about oncology drugs that were approved using either Accelerated Approval (FDA) or Notice of Compliance with conditions (NOC/c, Health Canada) and that failed confirmatory trials.

**Methods:**

Drug/indications approved by the FDA through its Accelerated Approval Pathway and that later failed confirmatory studies were identified from a published study and additional information on these drugs was collected from Drugs@FDA. Health Canada websites were searched on September 11, 2021 for the same group of drugs to determine if they were approved in Canada under the NOC/c pathway for the same indication as in the US. Information from both the FDA and Health Canada about these products was entered into an Excel spreadsheet. Decisions about whether to withdraw the drugs or remove the failed indication for the drug and requirements for confirmatory studies were compared. In addition, the dates of decisions by the two agencies were compared.

**Results:**

Ten drug/indications were available for comparison. Regulatory decisions were similar in 4 cases, different in 1 case and could not be determined in the remaining 5, in 1 case because decisions were pending in both countries and in the other 4, because the NOC/c had not been fulfilled in Canada. The requirements for the confirmatory studies were similar in both countries. Decisions were made earlier in the United States.

**Conclusions:**

This study shows that decisions made by Health Canada and the FDA about whether to withdraw a drug or remove a failed indication when drug/indications fail a confirmatory trial are usually similar, although the sample size on which this conclusion is made is small. The clinical implications of these similarities and differences should be explored.

**Supplementary Information:**

The online version contains supplementary material available at 10.1186/s40545-021-00375-y.

## Introduction

Differences between drug regulators in decisions about whether to authorize new drugs for marketing, the type of approval used and which indications to approve have been documented in the past for oncology drugs [1–3].

In the United States (US), the Food and Drug Administration (FDA) uses the Accelerated Approval pathway to conditionally approve new drugs based on surrogate markers when those products are reasonably likely to predict actual clinical benefit. Upon approval, companies commit to undertaking trials to confirm the preliminary conclusions about benefits [4]. Similar to the Accelerated Approval pathway, Health Canada has its Notice of Compliance with conditions (NOC/c) pathway [5]. Over the period 2016–2020, use of these pathways has been dominated by oncology drugs. In the US, 93 out of 108 Accelerated Approvals were for drugs with an oncology indication (https://www.fda.gov/media/151146/download), while in Canada 40 out of 47 drugs approvals through the NOC/c pathway were for drugs to treat cancer (https://www.canada.ca/en/health-canada/services/drugs-health-products/drug-products/notice-compliance/conditions.html).

One of the requirements of conditional approvals by both Health Canada and the FDA is that companies conduct additional trials to verify the efficacy of the products. If these confirmatory studies are negative, both regulatory agencies have the option to either withdraw the indication or, if the drug has only one indication to remove the drug from the market. To date, there has not been any investigation about the agreement or disagreement between the regulatory actions of different national drug agencies when confirmatory trials are negative.

The article by Gyawali and colleagues [6] examines regulatory decisions by the FDA following failed confirmatory trials of cancer drugs. This current study compares decisions made by Health Canada about these same drugs with those made by the FDA; specifically decisions about whether to allow the drug to remain on the market and/or to remove the failed indication for the drug. Secondarily, it also compares the requirements for the confirmatory trials by the two agencies, since different requirements might lead to different decisions. Finally, this study compares three time periods: differences in approval dates, differences in dates for decisions and differences in the length of time between the date of drug approvals and the date when decisions about drug/indications were made.

## Methods

### Information from the FDA and Health Canada

The article by Gyawali evaluated 18 oncology drug/indications approved by the FDA that failed confirmatory trials from the inception of the Accelerated Approval program in 1992 until December 2020. Health Canada websites (https://health-products.canada.ca/dpd-bdpp/index-eng.jsp; https://health-products.canada.ca/noc-ac/index-eng.jsp) were searched on September 11, 2021 for the same group of drugs to determine if they were approved in Canada under the NOC/c pathway for the same indication as in the US. Drugs that were approved in the two countries under conditional approval pathways for the same indication formed the sample to be investigated.

From the Gyawali article the following information about these drugs was extracted and entered in an Excel spreadsheet: generic drug name and decision about Accelerated Approval indication and the drug as of May 2021. Additional information about the drug approval date, i.e., date when marketing was authorized, requirements for the confirmatory trials and updates to September 11, 2021 about the status of the drug/indication along with the date of that decision was gathered from the label, approval letter and supplements available at Drugs@FDA (https://www.accessdata.fda.gov/scripts/cder/daf/index.cfm). The same information on this group of drugs (approval date, requirements for the confirmatory trials, status of the drug/indication and date of decision) was gathered from the Health Canada websites or from previously filed Access to Information requests.

### Analysis

Decisions about the status of drug/indications in the two countries along with requirements for the confirmatory trials were compared. Differences, in years, between approval dates and decision dates in Canada and the US were calculated. Differences in the length of time between approval and decision dates in Canada and the US were calculated and compared using a *t* test with a level of significance of *p* < 0.05. Calculations were done using Excel for Macintosh 16.53 (Microsoft Corporation).

### Ethics

All data were publicly available and ethics approval was not required.

## Results

Out of the 18 drug/indications reported by Gyawali, 5 drug/indications (27.8%) were not submitted for approval in Canada and 3 of the remaining 13 drug/indications (23.1%) were approved through other regulatory pathways. Ten drug/indications were available for comparison regarding the decisions made by the FDA and Health Canada following the failed trials, the requirements for the confirmatory trials, and the various time periods (Table 1). (All of the data collected is available in Additional file 1: Table S1.)

**Table 1:** Comparison of oncology drug/indication combinations approved by Food and Drug Administration and Health Canada requiring Canada

Generic name	Approved indication	Food and Drug Administration			Health Canada			Comparison of decisions
		Date of marketing approval	Decision about drug/indication	Date of decision about drug/indication	Date of marketing approval	Decision about drug/indication	Date of decision about drug/ indication	
Atezolizumab	PDL1 + triple negative breast cancer	2019-03-08	ODAC voted 7–2 against withdrawal	Pending as of 2021-09-11	2019-08-21	None	NOC/c not fulfilled as of 2021-09-11	Could not be determined
Atezolizumab	Urothelial cancer, second line	2016-05-18	Voluntarily withdrawn	2021-04-13	2017-04-12	None	NOC not fulfilled as of 2021-09-11	Could not be determined
Bevacizumab	Glioblastoma	2009-05-05	Converted to regular approval	2017-12-05	2010-03-24	Indication withdrawn, no reason given	2018-05-23	Different
Bevacizumab	HER2 negative breast cancer	2008-02-22	Revoked by FDA over objection of manufacturer	2011-12-20	2009-02-06	Indication suspended, no reason given	2011-12-25	Similar
Durvalumab	Urothelial cancer	2017-05-01	Voluntarily withdrawn	2021-02-19	2017-11-03	None	NOC not fulfilled as of 2021-09-11	Could not be determined
Gefitinib	Non-small cell lung cancer	2003-05-05	Voluntarily withdrawn; subsequently approved in different population (EGFR mutations positive only)	2005-06-17	2003-12-17	Converted to regular approval for patients who have activating mutations of the EGFR-TK	2009-12-18	Similar
Nivolumab	Hepatocellular cancer	2017-09-22	Voluntarily withdrawn	2021-07-23	2018-03-23	None	NOC not fulfilled as of 2021-09-11	Could not be determined
Nivolumab	Melanoma after ipilimumab or BRAF inhibitor	2014-12-22	Converted to regular approval on basis of results from other trials	2019-03-07	2016-04-29	Converted to regular approval	2018-02-21	Similar
Olaratumab	Soft tissue sarcoma	2016-10-19	Voluntarily withdrawn	2019-04-26	2017-11-23	Discontinued by company	2020-10-08	Similar
Pembrolizumab	Urothelial cancer, first line	2017-05-18	Converted to regular approval	2021-08-31	2019-04-11	None	2021-09-11	Could not be determined

*FDA*  Food and Drug Administration, *ODAC*  Oncology Drug Advisory Committee

Regulatory decisions were similar in 4 cases, different in 1 case and could not be determined in the remaining 5, in 1 case, because decisions were pending in both countries and in the other 4 because the NOC/c had not been fulfilled in Canada, i.e., either the confirmatory trial had not been submitted to Health Canada or it had not made a decision about whether the trial met the imposed requirements (Table 1). The requirements for the confirmatory studies were similar in both countries (Additional file 1: Table S1).

In all cases, the FDA approved the drug/indication before Health Canada, by a mean of 0.92 years (95% CI 0.59, 1.24). For the 5 drugs in both countries, where a decision had been made, those decisions came a mean of 1.08 years (95% CI 1.55, 3.71) earlier in the US although for nivolumab for the treatment of melanoma, a decision was made in Canada before it was made in the US. For the 4 drugs, where a decision had been made in the US but not in Canada, the time difference as of September 11, 2021, was a mean of 0.29 years (95% CI − 0.10, 0.67) (Table 2).

**Table 2:** Difference in dates of marketing approval and decisions about drug/indication between Canada and the United States

Difference in approval dates for all 10 drug/indications (years, 95% CI)*	Difference in decision dates about drug/indication for 5 drugs where both countries made decision (years, 95% CI)*	Difference in decision dates about drug/indication for 4 drugs where US made a decision and Canada had not (years, 95% CI)*
0.92 (0.59, 1.24)	1.08 (− 1.55, 3.71)	0.29 (− 0.10, 0.67)

*Time shorter in the US

The time from approval to decision for the 5 drugs with a decision in both countries was a mean of 4.25 years (95% CI 1.05, 7.45) in the US and 4.35 years (95% CI 1.06, 7.64) in Canada (*t* test, *p* = 0.9542).

## Discussion

This is the first study to compare regulatory decisions about drug/indications when approval was given under a conditional approval pathway and the drug subsequently failed the confirmatory trial. Congruence in the decisions could only be evaluated in 5 of the 10 cases and they were the same for 4 of the 5 drug/indications. Those decisions came slightly more than a year earlier in the US than in Canada. The inability to compare the decisions in 5 cases was because the NOC/c had not been fulfilled in 4 of these and in 1 case no decision had been made in both countries. In all 10 cases, the requirements for the confirmatory studies were very similar between those imposed by the FDA and by Health Canada. For the 4 drug/indications where a decision had not been made in Canada as of September 11, 2021, a decision had been made in the US 0.29 years earlier.

The requirements for confirmatory trials are set when a decision is made to approve a product either through the Accelerated Approval or the NOC/c pathway. The drugs in question in this study were approved almost a year earlier in the US, and since the confirmatory trial requirements set by the two agencies were quite similar, the Health Canada conditions should not have required the companies to start de novo, i.e., either planning for the trials or the trials themselves should have been underway. If this was the case, then it raises questions about why Canadian decision times were not shorter than those in the US. The longer Canadian times could be because of delays in company submission of the trial results, which is in line with previous work showing that companies submit drugs for approval to Health Canada a median of 340 days after they are submitted to the European Medicines Agency (EMA) or the FDA [7]. A second factor might be that the FDA was faster in making a decision about the confirmatory study, again in line with the difference in median regulatory time for Accelerate Approval drugs of 173 days compared to 253 days for NOC/c drugs [7].

This study adds to the literature showing both similarities and differences in regulatory decision making about oncology drugs. When Shah and colleagues compared decision making by the FDA and the EMA for tyrosine kinase inhibitors, they showed that the FDA gave a priority review to 12 of them but the EMA did not use its equivalent for any. On the other hand, the FDA used Accelerated Approval for 6 and the EMA used the equivalent conditional approval for 4. The active review and approval times in both agencies were quite similar [1]. Similarly, for oncology drugs more generally, most of the 21 drug/indications approved by both the FDA and the EMA between 2009 and 2013 relied on identical pivotal trials. At the same time, regulatory pathways often differed; 57% of indications received either FDA Accelerated Approval or EMA Conditional Marketing Authorization, and regular approval by the other agency [3]. Between 1995 and 2008, the EMA approved 42 oncology drugs for 100 indications and in 47 of these indications there was a discrepancy between the EMA and the FDA [2].

It is important to examine regulatory decisions about confirmatory trials when oncology drugs have been approved on the basis of surrogate outcomes. In about one-third (64 out of 194) oncology drug/indications approved by the FDA between 1992 and 2019, surrogate outcomes were used for the first time for a specific cancer type [8]. As Kemp and Prasad note “surrogates should lead to practice change or drug approval only when robust validation studies demonstrate that a change in a specific surrogate has a reliable ability to predict changes in meaningful outcomes.” [9]. However, this is often not the case. Improved patient survival was only demonstrated in confirmatory trials in 19 out of 93 oncology drug/indications approved by the FDA through its Accelerated Approval pathway between December 1992 and May 2017. Nineteen confirmatory trials reported improvement in the same surrogate used in the preapproval trial, and 20 reported improvement in a different surrogate [10]. In Canada, confirmatory trials for oncology drugs approved through the NOC/c pathway and that were accepted by Health Canada used surrogate outcomes in 11 out of 20 cases [11]. In light of these and other findings, Salcher-Konrad et al. raise the question about whether postmarketing studies imposed by regulators are “suited to deliver timely, confirmatory evidence due to shortcomings in study design and delays” and if conditional approval pathways are suitable “as tools for allowing early market access for cancer drugs while maintaining rigorous regulatory standards.”[3].

The main limitation to this study is the small number of drugs that were available for evaluation. Once a larger sample is available, further research should be done to see if the results remain the same. A second limitation is that all of the data was extracted and analyzed by one person.

## Conclusion

Oncology drugs are frequently conditionally approved based on surrogate outcomes and trials to confirm the benefits are necessary, but those trials can fail to show any benefit. In that case, regulators need to make a decision about whether to cancel either the indication or remove the drug from the market. This study shows that the decisions made by Health Canada and the FDA about whether to withdraw either the drug from the market and/or remove the indication in the face of failed trials are usually similar. The requirements for confirmatory trials are also similar, but the time to decisions is different with the US being faster. The clinical implications of these similarities and differences should be explored.

## Supplementary Information

**Additional file 1: Table S1.** Comparison of requirements for confirmatory studies.

## Data Availability

All the data is included in the manuscript.

## Abbreviations

EMA: European Medicines Agency
FDA: Food and Drug Administration
NOC/c: Notice of Compliance with conditions
US: United States
