# Supplementary material for: A comparison of the Food and Drug Administration’s and Health Canada’s regulatory decisions about failed confirmatory trials for oncology drugs: an observational study
Source: J Pharm Policy Pract. 2021 Oct 28;14:93. doi: 10.1186/s40545-021-00375-y (PMC8555114; doi:10.1186/s40545-021-00375-y)
Supplement: Supplementary file 1 — Additional file 1: Table S1. Comparison of requirements for confirmatory studies. [file 40545_2021_375_MOESM1_ESM.docx]

**Table S1: Comparison of requirements for confirmatory studies**

| **Generic name** | **Indication** | **Food and Drug Administration** | **Health Canada** |
| --- | --- | --- | --- |
| Gefitinib | Non-small cell lung cancer | 1. To conduct, submit, and publish the final study report for Protocol l 839IU0709 entitled "A randomized phase Ill survival study comparing ZD1839 (lressa TM"J plus best supportive care (BSC) versus placebo plus BSC in subjects with advanced NSCLC 2. To conduct, submit, and publish the final study report for a randomized trial comparing gefitinib and taxotere in NSCLC. 3. To conduct, submit, and publish the final study report for a randomized, controlled, double blind, study comparing ZD1839 treatment with best supportive care in refractory, symptomatic,   stage III/IV NSCLC patients (PS 0-2, LCS :S 20). | 1. To perform, complete and submit final data for Protocol 1839IL/0709. A double-blind, placebo controlled, parallel-group, multicentre, randomized, Phase III survival study comparing ZD1839 (IRESSA™) (250 mg tablet) plus best supportive care versus placebo plus best supportive care in patients with advanced NSCLC. 2. To perform, complete and submit final data for protocol 1839IL/0710. A double-blind, placebo-controlled, parallel-group, multicentre, randomized Phase III study of disease related symptoms comparing ZD1839 (IRESSA™) (250 mg tablet) plus best supportive care (BSC) versus placebo plus BSC in symptomatic patients with advanced NSCLC. 3. To perform, complete and submit final data for protocol 1839IL/0721. A randomized, open-label, parallel-group, Phase III trial of ZD1839 (IRESSA™) versus docetaxel. 4. To perform, complete and submit data for protocol 6474IL/0003. A phase II, randomized, double-blind, 2 part, multicenter study to compare the efficacy of ZD6474 with the efficacy of ZD1839 (IRESSA™) in subjects with locally advanced or metastatic (IIIB/IV) Non-Small Cell Lung Cancer |
| Bevacizumab | HER2 negative breast cancer | 1. Study BO 17708, "A Randomized, Double-Blind, P1acebo-Cnntrolled, Multicenter Study to Evaluate the Efficacy and Safety of Bevacizumåb in Combination with Docetaxe1 in Comparison with Docetaxe1Plus Placebo as First-Line Treatment or Patients with HER2-Negative Metastatic Breast Cancer." 2. AVF3694g "A Multicenter, Phase 3, Randomized, Placebo-Controlled Trial Evaluating the Efficacy and Safety of Bevacziumab in Combination with Chemotherapy Regimens in Subjects with Previously Untreated Metastatic Breast Cancer." | 1. AVADO (BO17708) is a phase III, double blind, placebo-controlled trial in which bevacizumab plus docetaxel chemotherapy was compared with docetaxel alone 2. RIBBON I (AVF3694g) is a phase III, placebo-controlled, blinded study in first-line metastatic HER-2 negative breast cancer investigating AVASTIN in combination with either taxane-based, anthracycline-based or XELODA (capecitabine) chemotherapies. |
| Bevacizumab | Glioblastoma | To submit an efficacy supplement containing the final study report…of study AVF4396g/BO20990 entitled “A Randomized, Double Blind, Placebo Controlled, Multicenter Phase III Trial of Bevacizumab, Temozolomide and Radiotherapy, Followed by Bevacizumab and Temozolomide Versus Placebo, Temozolomide Followed by Placebo and Temozolomide in Patients with Newly Diagnosed Glioblastoma. | BO21990 is a randomized, double-blind, placebo-controlled, multicenter Phase III trial of bevacizumab, temozolomide and radiotherapy, followed by bevacizumab and temozolomide versus placebo, temozolomide and radiotherapy followed by placebo and temozolomide in patients with newly diagnosed glioblastoma. |
| Nivolumab | Melanoma after ipilimumab or BRAF inhibitor | Conduct and submit the results of a multicenter, randomized trial or trials establishing the superiority of nivolumab over standard therapy in adult patients with unresectable or metastatic melanoma who are refractory to ipilimumab or who have not been previously treated with ipilimumab. | Results of a multicenter, randomized trial or trials establishing the superiority of nivolumab over standard therapy in adult patients with unresectable or metastatic melanoma who have disease  progression following ipilimumab and, if BRAF V600 mutation positive, a BRAF inhibitor…Bristol-Myers Squibb Canada has indicated that the final report of the completed trial CA209037 entitled "A Randomized, Open-Label Phase 3 Trial of BMS-936558 (Nivolumab) Versus Investigator's Choice in Advanced (Unresectable or Metastatic) Melanoma Patients  Progressing Post Anti-CTLA-4 Therapy" will be submitted to fulfil this commitment. |
| Atezolizumab | Urothelial cancer, second line | 1. Conduct "0029294: A Phase 3, Open-label, Multicenter, Randomized Study to Investigate the Efficacy and Safety of Atezolizumab Compared with Chemotherapy in Patients with Locally Advanced or Metastatic Urothelial Bladder Cancer After Failure with Platinum-containing Chemotherapy" 2. Develop and validate an assay with improved sensitivity for the detection of neutralizing antibodies against atezolizumab in the presence of atezolizumab levels that are expected to be present in samples at the time of patient sampling. Patient samples should be banked for storage until the improved method is available. 3. Conduct a clinical trial to evaluate the effect of atezolizumab on thyroid function tests and clinical thyroid disease. Submit the datasets with the completed report. 4. Submit the median duration of response for patients who responded to atezolizumab on 0029293 . This includes all patients and patients whose tumor infiltrating cells stain IC 2/3 or IC 0/1. Submit the datasets with the completed report. 5. Conduct an animal study that will measure the effect of PD-Ll inhibition on the magnitude of the primary (1st vaccination) and recall (2nd vaccination) antibody responses to antigen challenge (e.g., KLH). This study will evaluate the effect of PD-Ll inhibition on the primary immwie response once steady state plasma levels have been achieved and will reassess the magnitude of the recall response after a suitable period in the presence or absence of continued dosing. The study should include, if possible, an evaluation of cytokine production by T cells at appropriate time-points. | 1. Submit by the second quarter of 2018 (Q2 2018), as an SNDS-c, the final report for the primary analysis of the confirmatory study: A Phase III, open-label, multicenter, randomized study to investigate the efficacy and safety of atezolizumab compared with chemotherapy in patients with locally advanced or metastatic urothelial carcinoma after failure with platinum containing chemotherapy (IMvigor211). This study is designed to evaluate the effect of atezolizumab on overall survival (OS) when compared to chemotherapy (investigator's choice of docetaxel, paclitaxel or vinflunine) 2. Submit by the second quarter of 2018 (Q2 2018), as an SNDS-c, mature estimates of the secondary analyses (i.e., duration of response) of the pivotal study, IMvigor210 (Cohort 2). |
| Pembrolizumab | Urothelial cancer, first line | 1. Conduct clinical trial KEYNOTE-361 entitled “A Phase III Randomized, Controlled Clinical Trial of Pembrolizumab With or Without Platinum-Based Combination Chemotherapy Versus Chemotherapy in Subjects With Advanced or Metastatic Urothelial Carcinoma”. 2. Analyze the durability of response when all responders have had the opportunity for at least two years of follow-up in KEYNOTE-052 entitled “A Phase II Clinical Trial of Pembrolizumab (MK-3475) in Subjects with Advanced/Unresectable or Metastatic Urothelial Cancer”. | 1. Submit, as an SNDS-c, the final report for the confirmatory study: A phase 3, randomized, active controlled trial of pembrolizumab with or without platinum-based combination chemotherapy versus chemotherapy in subjects with advanced or metastatic urothelial carcinoma (KEYNOTE-361). 2. Submit, as an SNDS-c, the final report for the confirmatory study: A phase 3, randomized, double-blind study of pembrolizumab in combination with lenvatinib versus pembrolizumab and placebo as first line treatment for locally advanced or metastatic urothelial carcinoma in cisplatin-ineligible participants whose tumors express PDL1, and in participants ineligible for any platinum-containing chemotherapy regardless of PD-L1 expression (MK-7902-011). |
| Olaratumab | Soft tissue sarcoma | Conduct and submit the results of a multicenter, randomized clinical trial confirming the clinical benefit of olaratumab in combination with doxorubicin in patients with soft tissue sarcoma that is not amenable to surgery or radiation. | Submit, as an SNDS-C, the final clinical study report (CSR) for the confirmatory study JGDJ entitled “A Randomized, Double-Blind, Placebo-Controlled,  Phase III Trial of Lartruvo plus Doxorubicin versus Doxorubicin plus Placebo in Patients with Advanced or Metastatic Soft Tissue Sarcoma”. The SNDS-C  will be submitted by Q3/2020. |
| Atezolizumab | PDL1+ triple negative breast cancer | Submit the final progression free survival (PFS) analysis and datasets with the final report demonstrating clinical benefit of atezolizumab from clinical trial MO39196, IMpassion131, entitled; “A Study of Atezolizumab and Paclitaxel Versus Placebo and Paclitaxel in Participants With Previously Untreated Locally Advanced or Metastatic Triple Negative Triple Cancer (TNBC).” | Submit by December 2020, as an SNDS-c, the final overall survival analysis with the final report from the pivotal trial [study name removed] (i.e., according to the SAP, approximately 57 months after first patient in which was 23 June 2015). |
| Durvalumab | Urothelial cancer | 1. Submit the final report with datasets and labeling for the clinical trial entitled “A Phase III, Randomized, Open-label, Controlled, Multi-center, Global Study of First-line MEDI4736 Monotherapy and MEDI4735 in Combination with Tremelimumab Versus Standard of Care Chemotherapy in Patients with Unresectable Stage IV Urothelial Cancer.” 2. Conduct updated analyses of the duration of response for the patients with urothelial cancer who had received prior platinum-based therapy (N = 182) in the clinical trial entitled “A Phase 1-2 Study to Evaluate the Safety, Tolerability, and Pharmacokinetics of MEDI4736 in Subjects with Advanced Solid Tumors.” Present the median and updated information on the range of the duration of response for all patients, patients whose tumor have high PD-L1 staining, and patients whose tumors have low PD-L1 staining. Submit the final report with datasets and labeling. | 1. Submit, as an SNDS-c, the final report for the confirmatory study titled: A phase III, randomized, open-label, controlled, multi-center, global study of first-line MEDI4736 monotherapy and MEDI4736 in combination with tremelimumab versus standard of care chemotherapy in patients with unresectable stage IV urothelial cancer (D419BC00001). This study is designed to evaluate the effect, on overall survival, of durvalumab monotherapy and durvalumab in combination with tremelimumab vs. standard of care chemotherapy. 2. Submit, as an SNDS-c, mature estimates of the secondary analyses (i.e., duration of response) of the study, CD-ON-MEDI4736-1108, for patients   with UC.   1. AstraZeneca Canada Inc. should develop and validate assays with improved sensitivity for the detection of binding and neutralising antibodies against durvalumab in the presence of durvalumab concentrations that are expected to be present in samples at the time of patient sampling after first dose and during steady state. |
| Nivolumab | Hepatocellular cancer | 1. Conduct and submit the results, including datasets, of a multicenter, randomized trial or trials to verify and describe the clinical benefit of nivolumab over standard therapy based on an improvement in overall survival in patients with advanced hepatocellular carcinoma. 2. Submit the final report, including datasets, from patients with hepatocellular carcinoma who have progressed on, or are intolerant to sorafenib and who received nivolumab 3 mg/kg in the dose escalation or dose expansion phase of CHECKMATE-040. | 1. A Randomized, Multi-center Phase III study of Nivolumab versus Sorafenib as First-Line Treatment in Patients with Advanced Hepatocellular Carcinoma. This study shall need to verify the clinical benefits of nivolumab versus standard of care Sorafenib therapy based on an improvement of the overall survival (key primary endpoint) in patients with advanced hepatocellular carcinoma. A positive OS benefit shall be acceptable to fulfill the NOC/c confirmatory study requirement. 2. Final Report for hepatocellular carcinoma patients who have progressed on or are intolerant to sorafenib in the dose escalation and dose expansion phase. |
